# Possible Novel Nebovirus Genotype in Cattle, France

**DOI:** 10.3201/eid1706.100038

**Published:** 2011-06

**Authors:** Jérôme Kaplon, Eric Guenau, Philippe Asdrubal, Pierre Pothier, Katia Ambert-Balay

**Affiliations:** Author affiliations: Hôpital Universitaire de Dijon, Dijon, France (J. Kaplon, P. Pothier, K. Ambert-Balay);; Laboratoire Départemental de Côte d’Or, Dijon (E. Guenau, P. Asdrubal)

**Keywords:** viruses, norovirus, nebovirus, Nebraska bovine enteric calicivirus, cattle, zoonoses, epidemiology, zoonotic risk, France, dispatch

## Abstract

To determine if bovine caliciviruses circulate in France, we studied 456 fecal samples from diarrheic calves. We found a 20% prevalence of genogroup III noroviruses and a predominance of genotype III.2. Neboviruses, with a prevalence of 7%, were all related to the reference strain Bo/Nebraska/80/US, except for the strain Bo/DijonA216/06/FR, which could represent a novel genotype.

In the *Caliciviridae* family, genogroup III noroviruses (NoVsGIII) and neboviruses are associated with enteric disease in cattle (*1*), while genogroups I and II noroviruses (NoVsGI, NoVsGII) are a major cause of viral gastroenteritis in humans. Because of these common taxonomic and clinical features, molecular epidemiologic studies have been conducted on cattle worldwide to investigate possible zoonotic transmission. In France, little is known about the prevalence and genetic diversity of NoVsGIII and neboviruses circulating in cattle and possibly in humans.

## The Study

We collected 456 fecal samples from diarrheic calves (mean age 9 days, median 8 days) from 415 farms in Burgundy, France, during December 2005 through September 2008 to screen for these viruses: 1 sample each was collected for 377 outbreaks; 2 and 3 samples were collected for 35 and 3 outbreaks, respectively. Reverse transcription PCR, targeting the 3′ end of the polymerase gene of NoVsGIII and neboviruses, was done with the QIAGEN One Step RT-PCR kit (QIAGEN, Hilden, Germany), according to the manufacturer’s instructions, by using the primer sets CBECU-F/CBECU-R and NBU-F/NBU-R (*2*). The complete capsid gene of a selection of neboviruses was amplified by using NBcap-F3/NBcap-R primers (*3*). The amplified products of 532 bp, 549 bp, and 1,692 bp were sequenced by using the same primers.

In addition, we collected human stool samples in Burgundy and other regions of France during June 2006 through September 2008 and analyzed them for the presence of NoVsGIII and neboviruses. Samples included 60 samples from 21 gastroenteritis outbreaks of unknown etiology and 50 samples from 13 gastroenteritis outbreaks related to the consumption of oysters or water contaminated by human noroviruses and other enteric viruses.

Among the 456 samples from cattle, 114 (25%) were positive: 89 (20%) and 34 (7%) for NoVsGIII and neboviruses, respectively, with 9 (2%) samples infected by both viruses. These findings corresponded to 83 and 32 outbreaks positive for NoVsGIII and neboviruses, respectively, among which 9 presented co-infections in the same samples. The prevalence of NoVsGIII in similar studies range from 4% in the Netherlands (*4*) to 80% in Michigan, USA (*5*). These variations can be partly explained by differences in sampling strategies. The prevalence of neboviruses in our study was similar to that reported in other countries, e.g., 8% in the United Kingdom (*6*) and 9% in South Korea (*7*), but lower than in Ohio, USA (29%) (*2*). Furthermore, similar to our results, Smiley et al. (*2*) found a predominance of NoVsGIII compared to nebovirus, whereas in the United Kingdom and South Korea the prevalence of the 2 viruses was similar (*6**–**9*).

Sequencing and phylogenetic analyses of 89 NoVsGIII strains enabled them to be classified into 2 groups: 25 strains, representing 5% of the 456 specimens analyzed, were homologous to each other. They clustered with the genotype 1 reference strain Bo/Jena/80/DE on the gene fragment analyzed (Table 1, phylogenetic analyses not shown). The other 64 strains, 14% of the 456 samples, clustered with the genotype 2 reference strain Bo/Newbury2/76/UK (Newbury Agent [NA] 2). Partial polymerase sequences of a selection of these strains were submitted to the GenBank database under the accession nos. GU259570–GU259580 and FJ974131–FJ974136. The high number of sequences obtained in our study allowed us to highlight the existence of 2 distinct genotypes within NoVsGIII, as proposed by Ando et al. (*10*) and confirmed by others (*2**,**5**,**9*). Furthermore, the predominance of genotype 2 observed in our study is in keeping with numerous data (*2**,**4**,**8**,**9*). One study reported similar prevalence for the 2 genotypes, with a slight predominance of genotype 2 (*5*). All these results suggest that genotype 1 could be a minor circulating genotype and genotype 2 the main genotype worldwide.

**Table 1:** Genogroup III norovirus nt and aa identities calculated on the 3′ end polymerase*

Variable	Group 1, France			Group 2, France			Bo/Jena/80/DE (genotype 1)			Bo/Newbury2/76/UK (genotype 2)	
	nt identity	aa identity		nt identity	aa identity		nt identity	aa identity		nt identity	aa identity
Group 1 (25 strains)	80.4–100	94.6–100		71.3–78.0	84.6–90.4		83.4–90.8	96.4–100		71.7–76.8	85.6–88.5
Group 2 (64 strains)	71.3–78.0	84.6–90.4		83.6–100	97.6–100		72.0–76.1	85.6–88.5		82.8–91.9	97.6–100

*Identity expressed as a percentage calculated by using MEGA 4.0 (www.megasoftware.net). nt, nucleotide; aa, amino acid.

**Figure 1:**
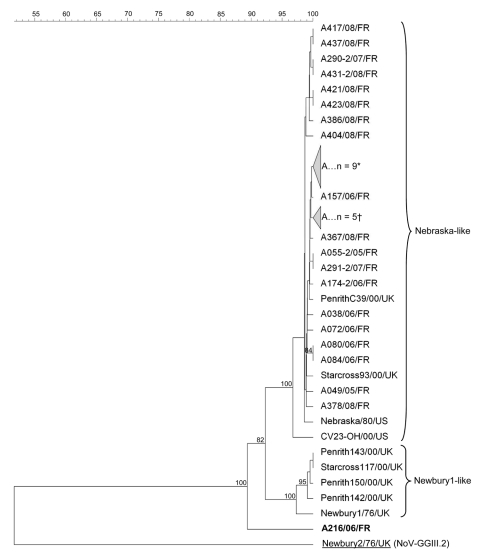
Nebovirus phylogenetic tree based on the deduced 167-aa–length sequences covering the 3′ end polymerase region. Possible novel strain is shown in **boldface**. Sequence alignments and clustering were performed by the unweighted-pair group method by using arithmetic average with Bionumerics software (Applied Maths, Sint-Martens-Latem, Belgium). Bootstrap values calculated with 1,000 replicate trees are given at each node when >70%. Collapsed branches are made up of the following nebovirus strains from France: *A042/06/FR, A051/05/FR, A058/05/FR, A130–2/06/FR, A143–2/06/FR, A311–2/08/FR, A381/08/FR, A445–2/08/FR, and A448/08/FR; †A281/07/FR, A355/07/FR, A356/07/FR, A364/08/FR, and A365/08/FR. Partial polymerase sequences of the French nebovirus strains were submitted to the GenBank database under accession nos. GU259537–GU259569 (A complete list is available from the authors.) GenBank accession nos. of calicivirus reference strains used in this tree are the following: AY082890 (CV23-OH/00/US), AY082891 (Nebraska/80/US), NC_007916 (Newbury1/76/UK), AF097917 (Newbury2/76/UK), DQ228162 (PenrithC39/00/UK), DQ228160 (Penrith142/00/UK), DQ228161 (Penrith143/00/UK), DQ228157 (Penrith150/00/UK), DQ228165 (Starcross93/00/UK), and DQ228164 (Starcross117/00/UK). Underlined strain is an outgroup. Scale bar indicates the percentage of similarities between strains.

Molecular and phylogenetic analyses of the partial polymerase region of 34 detected neboviruses revealed that 33 strains were homologous to each other and to the reference strain Bo/Nebraska/80/US (Nebraska strain [NB]) (*11*) (Table 2; Figure 1). No strain was related to the reference strain Bo/Newbury1/76/UK (NA1) (*1**,**6*). The same observation was made in the United States and South Korea, where only NB-like neboviruses were identified (*2**,**7*). A few studies have presented data about the epidemiology of neboviruses, but it seems that NA1-like neboviruses have only been identified in UK cattle (*1**,**6*). The 34th strain of our study, Bo/DijonA216/06/FR, was not related to NB or NA1 and appeared on a quite separate branch of the neboviruses.

**Table 2:** Nebovirus nucleotide and amino acid identities calculated on the 3′ end polymerase and capsid*

Variable	Nebovirus, France			Bo/Nebraska/80/US			Bo/Newbury1/76/UK	
nt identity	aa identity	nt identity	aa identity	nt identity	aa identity
Polymerase								
Nebovirus, France (33 strains)	88.4–100	96.0–100		87.9–90.2	96.6–98.0		76.8–79.5	87.9–89.3
Bo/DijonA216/06/FR strain	75.9–78.1	83.9–84.6		76.1	84.6		80.4	87.2
Capsid								
Nebovirus, France (6 strains)	85.3–97.7	92.9–99.6		85.2–91.1	92.9–99.0		84.6–92.3	92.9–99.2
Bo/DijonA216/06/FR strain	68.5–69.3	75.1–75.7		69.6	74.9		68.0	75.1

*Identity expressed as a percentage calculated by using MEGA 4.0 (www.megasoftware.net). nt, nucleotide; aa, amino acid.

**Figure 2:**
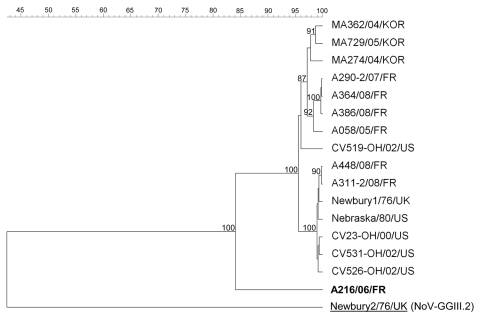
Nebovirus phylogenetic tree based on the deduced 549-aa–length sequences of the complete capsid. Possible novel strain is shown in **boldface**. Sequence alignments and clustering were performed by the unweighted-pair group method by using arithmetic average with Bionumerics software (Applied Maths, Sint-Martens-Latem, Belgium). Bootstrap values calculated with 1000 replicate trees are given at each node when >70%. Capsid sequences of the French nebovirus strains were submitted to the GenBank database under the following accession nos.: GU259542 (DijonA058/05/FR), FJ687386 (DijonA216/06/FR), GU259551 (DijonA290–2/07/FR), GU259553 (DijonA311–2/08/FR), GU259556 (DijonA364/08/FR), GU259561 (DijonA386/08/FR), and GU259569 (DijonA448/08/FR). GenBank accession nos. of Calicivirus reference strains used in this tree are: AY082890 (CV23-OH/00/US), AY549169 (CV519-OH/02/US), AY549170 (CV526-OH/02/US), AY549171 (CV531-OH/02/US), EF528561 (MA274/04/KOR), EF528564 (MA362/04/KOR), EF528569 (MA729/05/KOR), AY082891 (Nebraska/80/US), NC_007916 (Newbury1/76/UK), and AF097917 (Newbury2/76/UK). Underlined strain is an outgroup. Scale bar indicates the percentage of similarities between strains.

To gain more insight into the classification of neboviruses, we analyzed the complete capsid sequences of a selection of French neboviruses, including Bo/DijonA216/06/FR. While NB, NA1, and all but 1 of the French strains were closely related (Table 2; Figure 2), Bo/DijonA216/06/FR again presented low levels of identity with reference strains and appeared on a different branch on the phylogenetic tree. To our knowledge, there is no clear definition of a nebovirus genotype based on nucleotide or amino acid sequence identities. Oliver et al. (*6*) compared sequences of different NB-like and NA1-like strains and showed that strains of the same polymerase genotype presented >88% nt (>95% aa) identity in the polymerase region, while nucleotide sequence identities among strains from distinct polymerase type were <78% (88% aa) in this region. In a study from Korea, the minimum polymerase nucleotide identity between strains related to the NB-like viruses was 80.9% (84.5% aa), while these strains presented a maximum of 78.4% nt (82.8% aa) identity with NA1-like strains (*7*). Using capsid sequences, Oliver et al. (*6*) showed that nucleotide sequence identities within 1 type ranged from 92% to 94% (96% to 99% aa), whereas in Korea nucleotide sequence identities ranged from 85.7% to 87.7% (92.3 to 93.8% aa) (*7*). According to these data, Bo/DijonA216/06/FR appears to fall into a new genotype. This hypothesis is reinforced by the phylogenetic analysis of the amino acid sequences: Bo/DijonA216/06/FR clusters on a separate branch for both the polymerase and capsid regions, whereas the NB-like and NA1-like viruses cluster on separate branches in the polymerase region, but together in the capsid region. Given the capsid analyses, the new *Nebovirus* genus in the *Caliciviridae* family should thus comprise 2 genotypes: one would include the reference strains Bo/Nebraska/80/US and Bo/Newbury1/76/UK and the second would include Bo/DijonA216/06/FR. This hypothesis awaits confirmation, and the complete genome of this strain is now being characterized in our laboratory.

## Conclusions

The existence of animal reservoirs for human NoVs has been suggested (*4**,**8**,**12*) but, to our knowledge, only Mattison et al. (*13*) detected NoVsGII in fecal samples from pigs and cattle. In our study, we found no evidence of zoonotic transmission, since no NoVsGIII or neboviruses were detected in any samples from humans, which, for NoVsGIII, is consistent with the results of Wolf et al. (*14*). However, in contrast to these observations, Widdowson et al. (*15*) detected serum antibodies raised against NoVsGIII in humans, which suggests that NoVsGIII do infect humans.

This study found that NoVsGIII are more frequent than neboviruses in diarrheic calves in France. Our results confirmed the predominance of genotype 2 NoVsGIII, as previously reported (*2**,**4**,**8**,**9*), and showed that NB-like viruses are the major circulating strains of neboviruses. In addition, substantial genetic diversity of neboviruses was demonstrated with the existence of a possible novel strain, which could represent a new genotype.
